# Nicotinamide Effects Oxidative Burst Activity of Neutrophils in Patients with Poorly Controlled Type 2 Diabetes Mellitus

**DOI:** 10.1080/15438600490424244

**Published:** 2004

**Authors:** Zeynep Osar, Tülay Samanci, Gülderen Yanikkaya Demirel, Taner Damci, Hasan Ilkova

**Affiliations:** 1 Department of Internal Medicine Division of Endocrinology, Metabolism, and Diabetes Cerrahpasa Medical Faculty Istanbul University Istanbul Turkey; 2 Pakize Tarzi Flow Cytometry Laboratory Memorial Hospital Istanbul Turkey; 3 Havaci Feyza Sok. P26/9 Ataköy 3. Kisim Bakirköy Istanbul Turkey

## Abstract

Neutrophil functions are impaired in patients with diabetes mellitus. Bacterial phagocytosis and oxidative burst activity are reduced at high glucose concentrations in diabetic patients. Defects in neutrophil oxidative burst capacity are of multifactorial origin in diabetes mellitus and correlate with glucose levels. It has been reported that neutrophil NADPH oxidase activity is impaired and superoxide production is reduced in diabetic patients with or without any infections. Nicotinamide is a vitamin B3 derivative and a NAD precursor with immunomodulatory effects. In vitro studies demonstrated that nicotinamide increases NAD and NADH content of beta cells. The authors hypothesized that nicotinamide may restore the impaired oxidative burst capacity of neutrophils in diabetic patients by increasing the NADH content as an electron donor and possibly through NADPH oxidase activity of the cell. In order to test the hypothesis, this placebo-controlled and open study was designed to evaluate neutrophil functions in infection-free poorly controlled type 2 diabetic patients as compared to healthy subjects and assess the effects of nicotinamide on neutrophil phagocytosis as well as oxidative burst activity. Thirty patients with type 2 diabetes mellitus were enrolled in the study. Sixteen were females and 14 were males, with a mean age 58 ± 10. All patients were on sulphonylurea treatment and their hemoglobin A_1c_ (HbA_1c_) levels were above 7.5%. The control group consisted of 10 voluntary healthy subjects. Diabetic and control subjects were not significantly different in terms of age, body mass index (BMI), leucocyte and neutrophil counts, C-reactive protein (CRP) level, and erythrocyte sedimentation rate (ESR), but HbA_1c_ and fasting glucose levels were significantly higher in patients with diabetes mellitus. Phagocytic activity and respiratory burst indexes were measured by flow cytometric analyses as previously described by Rothe and Valet (*Methods Enzyml.*, 233, 539–548, 1994) and compared in diabetic subjects and healthy controls. Diabetic patients were grouped to receive either 50 mg/kg oral nicotinamide (n = 15) or placebo (n = 15) for a period of 1 month. The 2 groups did not differ in terms of treatment, frequency of hypertension, BMI, diabetes duration, age, fasting plasma glucose (FPG), HbA_1c_, CRP, ESR, polymorphonuclear leukocyte (PNL) and neutrophil counts. Neutrophil functions were reassessed after the treatment period. Phagocytic activity represented as indexes were lower in diabetic patients when compared to healthy subjects, but the differences were not statistically significant (*P* > .05). Patients with diabetes mellitus had significantly lower oxidative burst indexes when compared to healthy controls (*P* values < .05). In diabetic patients, a negative correlation between neutrophil functions and HbA_1c_ was found which was not statistically significant (*P* values > .05). Phagocytic indexes were similar in nicotinamide and placebo groups after treatment period (*P* > .05). But oxidative burst activity in patients receiving nicotinamide was greater when compared with placebo and the difference was statistically significant at 30 and 45 minutes (*P* values .04 and .03). This effect of nicotinamide may be due to increased NADH content and NADPH oxidase activity of the cell, which needs to be further studied. Impaired neutrophil functions may aggravate various infections in patients with diabetes mellitus and blood glucose regulation is an important target of treatment to improve neutrophil functions. But nicotinamide treatment may help to improve prognosis in diabetic patients with severe infections.


*Experimental Diab. Res.*, 5:155–162, 2004
Copyright © Taylor and Francis Inc.
ISSN: 1543-8600 print / 1543-8619 online
DOI: 10.1080/15438600490424244

Taylor & Francis
Taylor & Francis Group

### Nicotinamide Effects Oxidative Burst Activity of Neutrophils in Patients with Poorly Controlled Type 2 Diabetes Mellitus

**Zeynep Osar,[1] Tülay Samanci,[1] Gülderen Yanikkaya Demirel,[2] Taner Damci,[1] and Hasan Ilkova[1]**

[1]*Department of Internal Medicine, Division of Endocrinology, Metabolism, and Diabetes, Cerrahpasa Medical Faculty, Istanbul University, Istanbul, Turkey*
[2]*Pakize Tarzi Flow Cytometry Laboratory, Memorial Hospital, Istanbul, Turkey*

**Neutrophil functions are impaired in patients with diabetes mellitus. Bacterial phagocytosis and oxidative burst activity are reduced at high glucose concentrations in diabetic patients. Defects in neutrophil oxidative burst capacity are of multifactorial origin in diabetes mellitus and correlate with glucose levels. It has been reported that neutrophil NADPH oxidase activity is impaired and superoxide production is reduced in diabetic patients with or without any infections. Nicotinamide is a vitamin B3 derivative and a NAD precursor with immunomodulatory effects. In vitro studies demonstrated that nicotinamide increases NAD and NADH content of beta cells. The authors hypothesized that nicotinamide may restore the impaired oxidative burst capacity of neutrophils in diabetic patients by increasing the NADH content as an electron donor and possibly through NADPH oxidase activity of the cell. In order to test the hypothesis, this placebo-controlled and open study was designed to evaluate neutrophil functions in infection-free poorly controlled type 2 diabetic patients as compared to healthy subjects and assess the effects of nicotinamide on neutrophil phagocytosis as well as oxidative burst activity. Thirty patients with type 2 diabetes mellitus were enrolled in the study. Sixteen were females and 14 were males, with a mean age $58 \pm 10$. All patients were on sulphonylurea treatment and their hemoglobin $A_{1c}$ ($HbA_{1c}$) levels were above 7.5%. The control group consisted of 10 voluntary healthy subjects. Diabetic and control subjects were not significantly different in terms of age, body mass index (BMI), leucocyte and neutrophil counts, C-reactive protein (CRP) level, and erythrocyte sedimentation rate (ESR), but $HbA_{1c}$ and fasting glucose levels were significantly higher in patients with diabetes mellitus. Phagocytic activity and respiratory burst indexes were measured by flow cytometric analyses as previously described by Rothe and Valet (*Methods Enzyml.*, 233, 539–548, 1994) and compared in diabetic subjects and healthy controls. Diabetic patients were grouped to receive either 50 mg/kg oral nicotinamide (n = 15) or placebo (n = 15) for a period of 1 month. The 2 groups did not differ in terms of treatment, frequency of hypertension, BMI, diabetes duration, age, fasting plasma glucose (FPG), $HbA_{1c}$, CRP, ESR, polymorphonuclear leukocyte (PNL) and neutrophil counts. Neutrophil functions were reassessed after the treatment period. Phagocytic activity represented as indexes were lower in diabetic patients when compared to healthy subjects, but the differences were not statistically significant ($P > .05$). Patients with diabetes mellitus had significantly lower oxidative burst indexes when compared to healthy controls ($P$ values $< .05$). In diabetic patients, a negative correlation between neutrophil functions and $HbA_{1c}$ was found which was not statistically significant ($P$ values $> .05$). Phagocytic indexes were similar in nicotinamide and placebo groups after treatment period ($P > .05$). But oxidative burst activity in patients receiving nicotinamide was greater when compared with placebo and the difference was statistically significant at 30 and 45 minutes ($P$ values .04 and .03). This effect of nicotinamide may be due to increased NADH content and NADPH oxidase activity of the cell, which needs to be further studied. Impaired neutrophil functions may aggravate various infections in patients with diabetes mellitus and blood glucose regulation is an important target of treatment to improve neutrophil functions. But nicotinamide**

Received 15 January 2003; accepted 15 September 2003.
This study is supported by Istanbul University Research Fund.
Address correspondence to Zeynep Osar, Havaci Feyza Sok. P26/9, Ataköy 3. Kisim, Bakirköy, Istanbul, Turkey. E-mail: zeyneposar@yahoo.com

**treatment may help to improve prognosis in diabetic patients with severe infections.**

**Keywords** NADH; NADPH Oxidase; Neutrophils; Nicotinamide; Oxidative Burst Activity; Phagocytosis; Type 2 Diabetes Mellitus

In diabetic patients, polymorphonuclear leukocyte (PNL) functions are altered at different steps. Impaired chemotaxis, defective phagocytosis, and increased production of free radicals have been reported to occur in diabetes mellitus (DM) [1–3]. PNL oxidative burst activity and bacterial ingestion and killing are reduced at high glucose concentrations in diabetic patients [4]. These derrangements in neutrophil functions may increase the risk of infection in DM.

The oxidative burst is an important step in bacterial killing and involves a series of metabolic events that take place when phagocytes are stimulated, resulting in the production of superoxide ($O_2^-$), $H_2O_2$, and other more potent oxidizing radicals. These reactions are coupled with an increase in glucose oxidation via the hexose monophosphate shunt. Most of the oxidative burst is caused by activation of an NADPH oxidase that catalyses 1-electron reduction of oxygen to superoxide, using NAPDH as the electron donor [5]. Defects in neutrophil oxidative burst capacity are of multifactorial origin in diabetic patients and correlates with glucose levels [6]. Decreased NADPH levels in connection with reduced NADPH oxidase activity is one of the most underlined mechanisms in the impairment of PNL functions in DM. It has been reported that neutrophil NADPH oxidase activity is impaired and superoxide production is reduced in diabetic patients without any infection [6] as well as with periodontitis [7] or foot infections [8].

Nicotinamide is a vitamin B3 derivative and a NAD precursor with immunomodulatory effects. It has been proposed that nicotinamide supresses poly(ADP-ribose)polymerase (PARP) activity and, to a lesser extent, (mono)ADP-ribosyl tranferase activity [9, 10]. Supression of PARP activity decreases consumption of NAD, the substrate for PARP [10]. In vitro studies demonstrated that nicotinamide increases NAD and NADH content of beta cells [11].

The physiological electron donor of respiratory burst oxidase is NADPH, but the enzyme is also capable of using NADH, though less efficiently [5]. We hypothesized that nicotinamide may restore the impaired oxidative burst capacity of neutrophils in diabetic patients by increasing the NADH content and possibly through NADPH oxidase activity of the cell. In order to test the hypothesis, this study was designed to evaluate neutrophil functions in infection-free poorly controlled type 2 diabetic patients as compared to healthy subjects and assess the effects of nicotinamide both on neutrophil phagocytosis and as oxidative burst activity. The study is designed as a single blind study.

**TABLE 1**
Baseline characteristics of diabetic patients and controls

| | Type 2 DM (mean ± SD) | Control (mean ± SD) | *P* value |
|---|---|---|---|
| N | 30 | 10 | |
| Gender (F/M) | 16/14 | 5/5 | |
| Age (years) | 58 ± 10 | 58 ± 9 | .924 |
| Duration of DM (years) | 5 ± 3 | — | |
| BMI (kg/m$^2$) | 29 ± 5 | 27 ± 4 | .192 |
| FPG (mg/dL) | 173 ± 34 | 91 ± 12 | .0001* |
| $HbA_{1c}$ (%) | 8.7 ± 1.1 | 5.0 ± .3 | .0001* |
| CRP (mg/L) | 2.7 ± .9 | 2.6 ± .6 | .214 |
| ESR (mm/h) | 22 ± 11 | 19 ± 9 | .453 |
| PNL (/mm$^3$) | 7370 ± 1448 | 6900 ± 1575 | .215 |
| Neutrophil (/mm$^3$) | 4536 ± 1089 | 4025 ± 907 | .312 |

*Statistically significant.

### MATERIALS AND METHODS

### Subjects

Thirty patients with type 2 DM were enrolled in the study. Sixteen were females and 14 were males, with an age of 58 ± 10 years (mean ± SD), ranging between 44 and 75 years. The mean duration of diabetes was 5.0 ± 3.0 years (mean ± SD) (range 1 to 10 years). All patients were on sulphonylurea treatment (gliclazide or glipizide) and their hemoglobin $A_{1c}$ ($HbA_{1c}$) levels were above 7.5%. The control group consisted of 10 voluntary healthy subjects. Baseline characteristics of study subjects and controls were as in Table 1. There was no age and body mass index (BMI) difference between diabetic patients and healthy controls. Exclusion criteria were pregnancy, systemic glucocorticoid treatment, systemic infection, liver function tests greater than 1.5 times of normal, serum creatinine greater than 1.2 mg/dL, urinary albumin excretion greater than 200 $\mu$g/min, proliferative diabetic retinopathy, malignancy, and antiinflammatory or immunosuppressive treatment. The study protocol was in accordance with Helsinki Declaration and approved by the local ethical committee. All patients gave written informed consent.

### Initial Assessment

At the beginning of the study, clinical examination, leukocyte and neutrophil counts, and erythrocyte sedimentation rate (ESR), quantitative C-reactive protein (CRP), fasting plasma glucose (FPG), and $HbA_{1c}$ measurements were performed. Diabetic and control subjects were not significantly different in terms of PNL and neutrophil counts, CRP level, and ESR, but $HbA_{1c}$ and FPG levels were significantly higher in patients

**TABLE 2**
Baseline characteristics of diabetic patients receiving nicotinamide and placebo

| | Nicotinamide (mean ± SD) | Placebo (mean ± SD) | *P* value |
|---|---|---|---|
| N | 15 | 15 | |
| Gender (F/M) | 8/7 | 8/7 | |
| Age (years) | 55 ± 10 | 59 ± 8 | .435 |
| Duration of DM (years) | 5 ± 2 | 4 ± 3 | .973 |
| BMI (kg/m$^2$) | 30 ± 5 | 28 ± 3 | .156 |
| FPG (mg/dL) | 173 ± 30 | 173 ± 39 | .958 |
| $HbA_{1c}$(%) | 9.0 ± 1.2 | 8.4 ± 1.0 | .136 |
| CRP (mg/L) | 2.0 ± .9 | 2.2 ± .6 | .312 |
| ESR (mm/h) | 22 ± 11 | 19 ± 9 | .453 |
| PNL (/mm$^3$) | 7633 ± 1412 | 7106 ± 1484 | .328 |
| Neutrophil (/mm$^3$) | 4912 ± 1074 | 4160 ± 999 | .057 |

with DM (Table 1). Phagocytic activity and respiratory burst indexes were measured and compared in diabetic subjects and healthy controls.

### Nicotinamide Administration

Diabetic patients were grouped to receive either oral nicotinamide (n = 15) or placebo (n = 15) for a period of 1 month. The 2 groups did not differ in terms of diabetes treatment, frequency of hypertension, BMI, diabetes duration, age, FPG, $HbA_{1c}$, CRP, ESR, or PNL and neutrophil counts (Table 2). Nicotinamide was administered 50 mg/kg divided in three doses taken 30 minutes before meals. Clinical examination, PNL and neutrophil counts, and CRP, FPG, and $HbA_{1c}$ measurements were repeated and neutrophil functions were reassessed after the treatment period. Patients were questioned about possible side effects of nicotinamide.

### Biochemical and Hematological Analyses

Fasting venous plasma glucose was measured by glucose oxidase method. $HbA_{1c}$ determination was based on the turbidimetric inhibition immunoassay of hemolyzed whole blood (Roche) (normal range 2.5% to 4.5%). CRP was quantitatively measured by turbidimetric method (Behring) (normal range: 0 to 5 mg/L). ESR was measured manually in citrated whole blood. PNL and neutrophils were counted in Beckman Coulter Hmx Hematology Analyser.

### Assessment of Neutrophil Functions

After overnight fasting, 8 mL blood sample was taken from forearm vein into a tube containing 3 mL of density-gradient solution (Histopaque 1077, Sigma). After 40 minutes of gravity separation at room temperature, 800 $\mu$L of supernatant containing the leukocyte population was drawn out with plastic Pasteur pipette.

Phagocytosis and oxidative burst were measured by flow cytometric analyses as previously described by Rothe and Valet [12, 13]. Three tubes were prepared for each patient: for control, phagocytosis measurements and oxidative burst measurements. In each tube, 1 mL buffer solution (phosphate-buffered saline [PBS] containing 2% bovine serum albumin [BSA]), 20 $\mu$L leukocyte suspension and 5 $\mu$L Rhodamine 123 (end concentration 1 $\mu$g/mL) (Molecular Probes, USA) were

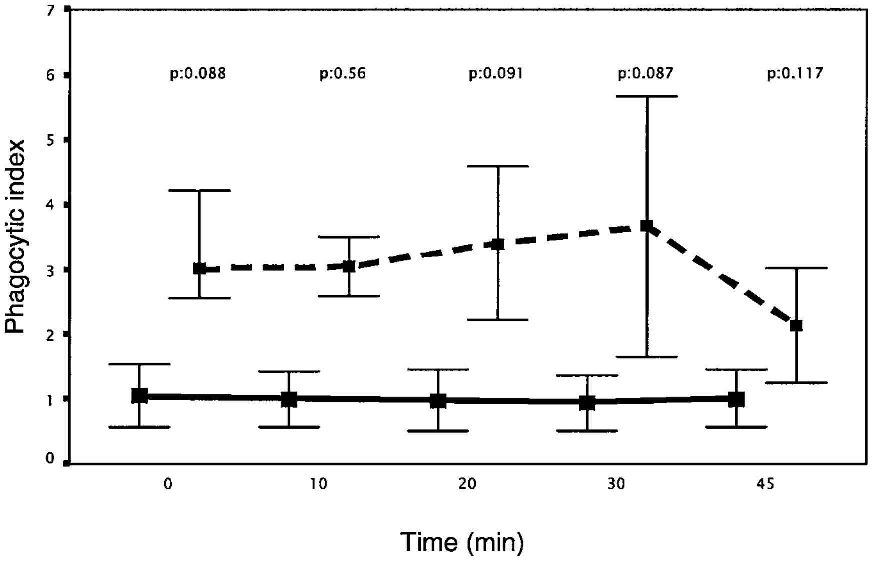

**FIGURE 1**
Initial bacterial phagocytosis as weighted phagocytic index in patients with DM (*solid line*) and controls (*dashed line*).

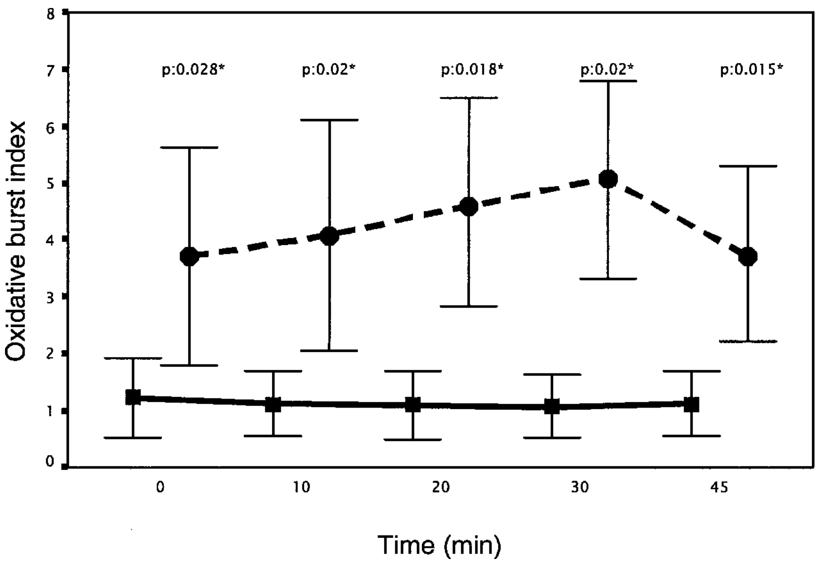

**FIGURE 2**
Initial oxidative burst indexes in patients with DM (*solid line*) and controls (*dashed line*). *Statistically significant.

mixed. Oxidative burst was stimulated with 10 $\mu$L phorbol myristate acetate (PMA; Sigma). *Staphyloccocus aureus* was used for assessment of phagocytosis. Flow cytometric analyses (Coulter EPICS XL-MCL) were performed at 0, 10, 20, 30, and 45 minutes. Phagocytosis and oxidative burst indexes were calculated by dividing the test mean channel numbers (flourescence density values) by the control mean channel numbers.

### Statistical Analyses

Independent-sample $t$ test for between-group comparisons and paired-sample $t$ test for in-group comparisons were performed using SPSS 10.0 for Windows. Correlations were assessed using Spearman's correlation coefficient. Statistical significance was accepted as $P < .05$.

### RESULTS

In diabetic subjects and healthy controls, phagocytic indexes positively correlated with oxidative burst indexes at 20, 30, and 45 minutes (correlation coefficients .35, .39, .43 and $P$ values .034, .036, and .021, respectively). Phagocytic activity represented as indexes were lower in diabetic patients when compared to healthy subjects, but the differences were statistically not significant ($P > .05$ at all time points) (Figure 1). Patients with DM had significantly lower oxidative burst indexes when compared to healthy controls at all time points ($P < .05$) (Figure 2). In diabetic patients, a negative correlation between neutrophil functions and $HbA_{1c}$ was found, which was not statistically significant ($P > .05$).

FPG, $HbA_{1c}$, CRP, and ESR levels as well as PNL and neutrophil counts were similar in nicotinamide and placebo groups at baseline (Table 2). No significant differences in baseline phagocytic (Figure 3) and oxidative burst indexes (Figure 4) were found in patients receiving nicotinamide and placebo ($P > .05$).

At the end of the treatment period, $HbA_{1c}$ levels decreased in both nicotinamide and placebo groups compared to baseline but the differences did not reach statistical significance ($P$ values .330 and .068, respectively). There were also no significant differences between nicotinamide and placebo groups in terms of FPG, $HbA_{1c}$, CRP, and ESR measurements, and PNL and neutrophil counts (Table 3).

Within the nicotinamide and placebo groups, we did not observe any significant difference when we compared phagocytic

**TABLE 3**
Measurements of diabetic patients after nicotinamide treatment

| | Nicotinamide (mean ± SD) | Placebo (mean ± SD) | $P$ value |
|---|---|---|---|
| N | 15 | 15 | |
| BMI (kg/m$^2$) | 30 ± 5 | 28 ± 3 | .156 |
| FPG (mg/dL) | 168 ± 59 | 177 ± 55 | .702 |
| $HbA_{1c}$(%) | 8.4 ± 1.6 | 8.1 ± 1.4 | .527 |
| CRP (mg/L) | 2.2 ± 1.0 | 2.4 ± .6 | .421 |
| ESR (mm/h) | 27 ± 12 | 22 ± 12 | .335 |
| PNL (/mm$^3$) | 7974 ± 2623 | 7093 ± 1506 | .269 |
| Neutrophil (/mm$^3$) | 5243 ± 1247 | 4550 ± 989 | .062 |

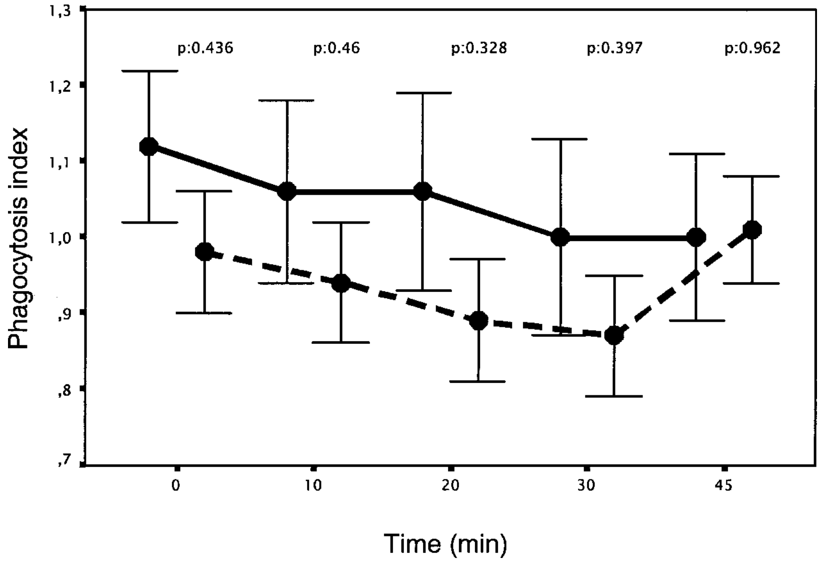

FIGURE 3
Initial phagocytic index levels in nicotinamide (*solid line*) and placebo groups (*dashed line*).

and oxidative burst indexes before and after the treatment (all $P > .05$). Phagocytic indexes were similar in nicotinamide and placebo groups after treatment period ($P > .05$ at all time points) (Figure 5). Oxidative burst activity in patients receiving nicotinamide was greater when compared with placebo; the difference, however, being statistically insignificant before the treatment became stastistically significant after nicotinamide treatment at 30 and 45 minutes ($P$ values .04 and .03, respectively) (Figure 6).

As far as side effects were concerned, nausea without vomitting was observed in 25% of nicotinamide group, but this symptom was limited and did not necessitate withdrawal of the drug in any case. We did not observe any side effects in the placebo group.

### DISCUSSION

The results of our study indicate that neutrophil functions are impaired in infection-free poorly controlled type 2 diabetic

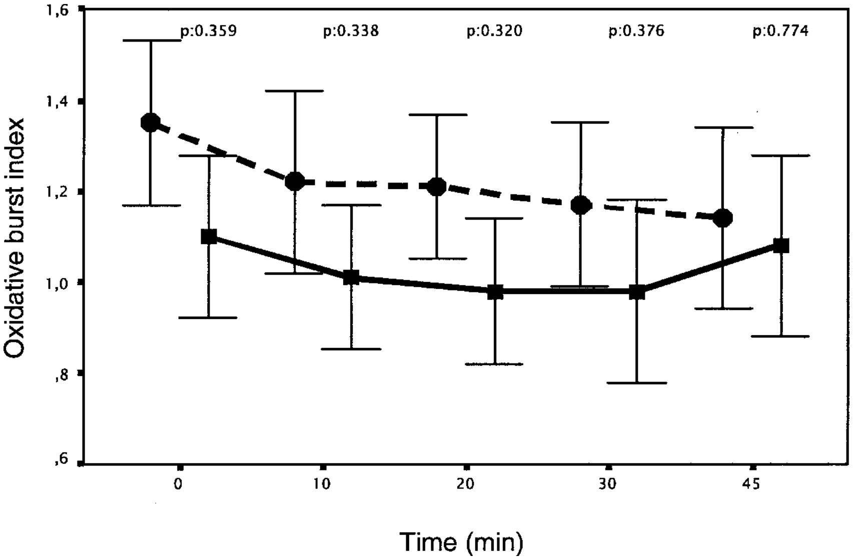

FIGURE 4
Initial oxidative burst index in nicotinamide (*dashed line*) and placebo (*solid line*) groups.

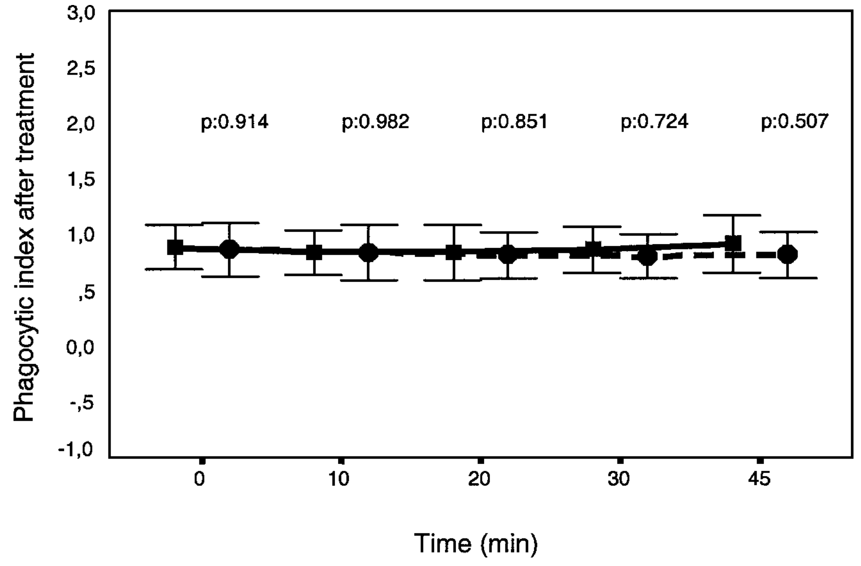

**FIGURE 5**
Phagocytic index after treatment in nicotinamide (*solid line*) and placebo (*dashed line*) groups.

patients. Phagocytosis and respiratory burst indexes tended to be lower in diabetic patients as compared to healthy controls, but the difference was only statistically significant for oxidative burst functions. Impaired PMA-induced respiratory burst activity and reduced phagocytosis of *S. aureus* have been reported in other studies in poorly controlled diabetic patients without any infections [4, 6]. A study using flow cytometric analyses as in our study showed lower PMA-induced respiratory burst activity in diabetic patients and results negatively correlated with $HbA_{1c}$ levels [6]. In our diabetic subjects we also observed a negative correlation between neutrophil functions and $HbA_{1c}$ levels, although this was not statistically significant.

It has been reported that neutrophil superoxide is significantly reduced during hyperglycemia in patients with type 2 DM [14]. Superoxide production is largely dependent on the activation of membrane-bound NADPH oxidase, which is an FAD-requiring enzyme using NADPH as the main physiological electron donor [5]. Therefore, reduced intracellular levels

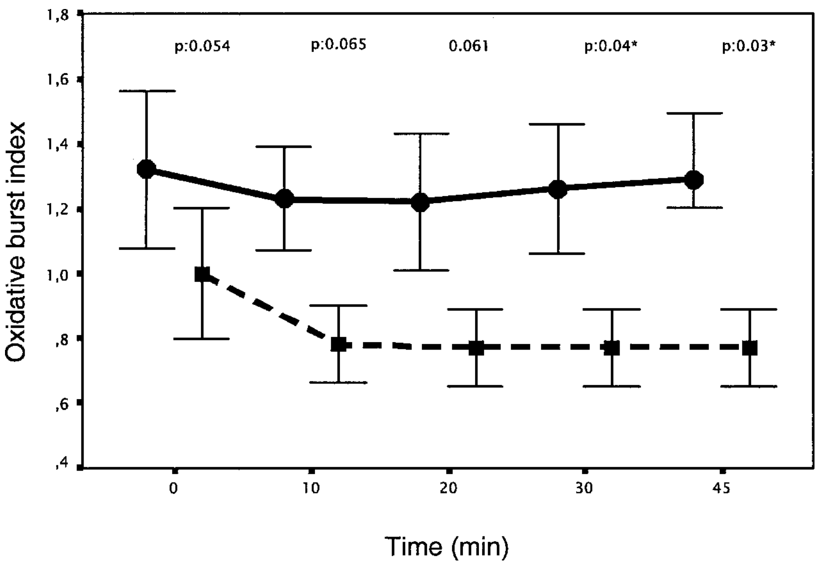

**FIGURE 6**
Oxidative burst index after treatment in nicotinamide (*solid line*) and placebo (*dashed line*) groups. *Significant.

of NADPH results in reduction of neutrophil superoxide production during respiratory burst [6, 7, 14]. NADPH oxidase is also capable of using NADH as an electron donor, although in a less efficient way [5].

We hypothesized that nicotinamide, as an NAD precursor, may restore impaired oxidative burst activity in neutrophils of diabetic subjects. In our diabetic patients treated with nicotinamide, oxidative burst functions were significantly higher at 30 and 45 minutes as compared to the placebo group, although the difference was not significant in the beginning and the end of the treatment. Because both groups of patients had similar characteristics, which otherwise could potentially influence PNL functions both at baseline and at the end of the study period, the difference in oxidative burst activity between the 2 groups suggests that the difference stems from the effect of nicotinamide treatment.

Nicotinamide supresses PARP activity, which leads to decreased consumption of NAD, the substrate for PARP [9, 10]. NADP differs from NAD only by phosphorylation of the C-2′ OH group on the adenosyl moeity. It has been shown that nicotinamide increases NAD + NADH content of beta cells and counteracts the fall of superoxide dismutase level in diabetic NOD mice [11], which may possibly be related to increased NAD content. The drug may restore the impaired oxidative burst capacity of neutrophils in diabetic patients, also by increasing the NADP content and by increasing the NADH consumption of the enzyme and NADPH oxidase activity of the cell. This effect may result in increased $H_2O_2$ synthesis and production of other more potent oxidizing radicals, such as oxidized halogens [15], and increase respiratory burst potential following nicotinamide treatment.

Most commonly observed side effects of nicotinamide are nausea and vomiting. It has been reported that 48% of patients taking 60 mg/kg nicotinamide experienced nausea and 32% vomitting [16]. Ruddock and colleagues showed that in isolated rat ileum, nicotinamide reduces the peristalsis by interaction with smooth muscle cells in a dose-dependent manner, which may be a reason for these symptoms [17]. In our study, only 25% of patients had nausea without vomiting and none discontinued treatment because of side effects.

Although the data showed negative effects of nicotinamide on insulin resistance [18], the nicotinamide group in the study showed no deterioration of metabolic control during the study.

In conclusion, neutrophil phagocytosis and respiratory burst capacity are impaired in poorly controlled diabetic patients as compared to healthy subjects and the differences in respiratory burst activity were significant between 2 groups. Nicotinamide administration at 50 mg/kg/day resulted in significant differences in respiratory burst activity at 30 and 45 minutes independent of blood glucose control. This finding may be due to nicotinamide effect, which needs be confirmed with larger studies. Impaired neutrophil functions may aggravate various infections in patients with DM and blood glucose regulation is an important target of treatment to improve neutrophil functions [6]. Nicotinamide treatment may also be of value as an adjunctive therapy.

### REFERENCES

[1] Delamaire, M., Maugendre, D., Moreno, M., LeGoff, M. C., Allannic, H., and Genetet, B. (1997) Impaired leucocyte functions in diabetic patients. *Diabet. Med.,* **14,** 29–34.

[2] Davidson, N. J., Sowden, J. M., and Fletcher, J. (1984) Defective phagocytosis in insulin controlled diabetics: Evidence for a reaction between glucose and opsoning proteins. *J. Clin. Pathol.,* **37,** 783–786.

[3] Sato, N., Shimizu, H., Suwa, K., Shimomura, Y., Kobayashi, I., and Mori, M. (1992) MPO activity and generation of active $O_2$ species in leucocytes from poorly controlled diabetic patients. *Diabetes Care,* **15,** 1050–1052.

[4] Marhoffer, W., Stein, M., Schleinkofer, L., and Federlin, K. (1993) Evidence of ex vivo and in vitro impaired neutophil oxidative burst and phagocytic capacity in type 1 diabetes mellitus. *Diabetes Res. Clin. Pract.,* **19,** 183–188.

[5] Babior, B. M. (1984) The respiratory burst of phagocytes. *J. Clin. Invest.,* **73,** 599–601.

[6] Ihm, S. H., Yoo, H. J., Park, S. W., and Park, C. J. (1997) Effect of tolrestat, an aldose reductase inhibitor, on neutrophil respiratory burst activity in diabetic patients. *Metabolism,* **46,** 634–638.

[7] De Toni, S., Piva, E., Lapolla, A., Fontano, G., Fidele, D., and Plebani, M. (1997) Respiratory burst of neutrophils in diabetic patients with periodontal disease. *Ann. N.Y. Acad. Sci.,* **832,** 362–367.

[8] Peck, K. R., Son, D. W., Song, J.-H., Sungmin, K., Oh, M.-D., and Choe, K. W. (2000) Enhanced neutrophil functions by recombinant human granulocyte colony-stimulating factor in diabetic patients with foot infections in vitro. *J. Korean Med. Sci.,* **16,** 39–44.

[9] Yamada, K., Nonaka, K., Hanafusa, T., Miyazaki, A., and Toyoshima, H. (1982) Preventive and therapeutic effects of large-dose nicotinamide injections on diabetes associated with insulitis. *Diabetes,* **31,** 749–753.

[10] Kolb, H., and Burkart, V. (1999) Nicotinamide in type 1 diabetes, mechanisms of action revisited. *Diabetes Care*, **22(Suppl 2),** B16–B20.

[11] Papaccio, G., Ammendola, E., and Pisanti, F. A. (1999) Nicotinamide decreases MHC class II but not class I expression and increases intercellular adhesion molecule-1 structures in non-obese diabetic mouse pancreas. *J. Endocr.,* **160,** 389–400.

[12] Rothe, G., Emmendörffer, A., Oser, A., Roesler, J., and Valet, G. (1991). Flow cytometric measurement of the respiratory burst activity of phagocytes using dihydrorhodamine 123. *J. Immunol. Methods,* **138,** 133–135.

[13] Rothe, G., and Valet, G. (1994) Flow cytometric assays of oxidative burst activity in phagocytes. *Methods Enzymol.,* **233,** 539–548.

[14] Mazade, M. A., and Edwards, H. M. S. (2001) Impairment of type III group B *Streptococcus*-stimulated superoxide production and opsonophagocytosis by neutrophils in diabetes. *Mol. Genet. Metab.,* **73,** 259–267.

[15] Babior, B. M. (1984) The respiratory burst of phagocytes. *J. Clin. Invest.,* **73,** 599–603.

[16] Bussink, J., Stratfort, M. R. L., van der Kogel, A., Folkes, L. K., and Kaanders, J. H. A. M. (2002) Pharmacology and toxicity of nicotinamide combined with domperidone during fractional radiotherapy. *Radiother. Oncol.,* **63,** 285–291.

[17] Ruddock, M. W., Burns, D. M., Murphy, L. E., O'Rourke, M. G., and Hirst, D. G. (2000) The effect of nicotinamide on spontaneous and induced activity in smooth and skeletal muscle. *Radiother. Oncol.,* **56,** 253–257.

[18] Greenbaum, C. J., Kahn, S. E., and Palmer, J. P. (1996) Nicotinamides effects on glucose metabolism in subjects at risk for IDDM. *Diabetes,* **45,** 1631–1634.

